# Assessing the Assessment in Emergency Care Training

**DOI:** 10.1371/journal.pone.0114663

**Published:** 2014-12-18

**Authors:** Mary E. W. Dankbaar, Karen M. Stegers-Jager, Frank Baarveld, Jeroen J. G. van Merrienboer, Geoff R. Norman, Frans L. Rutten, Jan L. C. M. van Saase, Stephanie C. E. Schuit

**Affiliations:** 1 Erasmus University Medical Center Rotterdam, Rotterdam, the Netherlands; 2 Training institution for family practice SBOH, Utrecht, the Netherlands; 3 McMaster University, Hamilton, Canada; 4 Maastricht University School of Health Professions Education, Maastricht, the Netherlands; Georgetown University Medical Center, United States of America

## Abstract

**Objective:**

Each year over 1.5 million health care professionals attend emergency care courses. Despite high stakes for patients and extensive resources involved, little evidence exists on the quality of assessment. The aim of this study was to evaluate the validity and reliability of commonly used formats in assessing emergency care skills.

**Methods:**

Residents were assessed at the end of a 2-week emergency course; a subgroup was videotaped. Psychometric analyses were conducted to assess the validity and inter-rater reliability of the assessment instrument, which included a checklist, a 9-item competency scale and a global performance scale.

**Results:**

A group of 144 residents and 12 raters participated in the study; 22 residents were videotaped and re-assessed by 8 raters. The checklists showed limited validity and poor inter-rater reliability for the dimensions “correct” and “timely” (ICC = .30 and.39 resp.). The competency scale had good construct validity, consisting of a clinical and a communication subscale. The internal consistency of the (sub)scales was high (α = .93/.91/.86). The inter-rater reliability was moderate for the clinical competency subscale (.49) and the global performance scale (.50), but poor for the communication subscale (.27). A generalizability study showed that for a reliable assessment 5–13 raters are needed when using checklists, and four when using the clinical competency scale or the global performance scale.

**Conclusions:**

This study shows poor validity and reliability for assessing emergency skills with checklists but good validity and moderate reliability with clinical competency or global performance scales. Involving more raters can improve the reliability substantially. Recommendations are made to improve this high stakes skill assessment.

## Introduction

### Background

Worldwide, over 1.5 million health care professionals attend a variety of certified emergency care courses each year, in which they are trained for the initial assessment and management of seriously ill patients, using a standardized approach [1]. At the end of most of these advanced emergency care courses, participants are assessed on their knowledge, typically using a multiple-choice test, and on their skills, typically using a single scenario test with a simulated patient and 1 or 2 raters, depending on the course, the country and the institution [2], [3].

The development of competencies in emergency care is a core component in both undergraduate and postgraduate medical curricula [1]. As emergency care skills are critical for doctors in emergency departments and hospitals, as well as for family practitioners, the quality of assessment is essential. It is vital that certification of such skills is based on robust testing, giving the certificate credibility [4]. Considering the widespread use of emergency care courses and assessment, the implications for patient safety and the high costs involved, it is surprising how little research is available on the quality of these assessments; in particular, their reliability and validity. A small number of studies have been done on advanced life support courses, sometimes showing poor inter-rater agreement [5] and sometimes showing moderate to good inter-rater agreement with small selected samples of participants [3]. Most studies recommend further research, but few provide specific recommendations. In the current study, we conduct psychometric analyses on a checklist, a 9-item competency scale and a global performance scale, used in a certified emergency care course. These assessment formats are commonly used in emergency care and other settings [6], [7].

### Purpose of this study

The purpose of this study was to assess the validity and reliability of commonly used formats (checklists, competency and global performance scales) in the assessment of emergency care skills for residents, and to make specific recommendations for further validation of these instruments.

## Methods

### Participants

Since 2009, emergency care courses are mandatory for emergency staff in all Dutch hospitals. Medical graduates are trained in standard emergency care methods. All Dutch family-practice residents are required to do a 6 months traineeship in an emergency department. Prior to the start all residents must complete a 2 week general emergency care preparatory course according to specific guidelines [8]. After passing this course they are allowed to start their traineeship under supervision of certified attending physicians. This course includes emergency care subjects such as the ABCDE-approach to emergency resuscitation. Each year 500–600 family-practice residents are trained and assessed, using a scenario assessment and knowledge test. The scenario assessment takes 15 minutes and is performed with one scenario, a standardized (trained) patient and one trained rater. This is a high stakes assessment for the residents: if they fail, one resit is offered with another scenario and two raters (including the course director). If a resident fails again, he/she is not allowed to start the emergency department traineeship.

All trainees in the present study were 2^nd^ year residents in the December 2012 or March 2013 course (n = 179). They were asked to consent to participate in the study and to being video-taped during their assessment. Fifteen raters with different medical backgrounds assessed the participants (one rater per candidate).

### Materials

The assessment instrument under evaluation aims to measure the skills and competencies of a doctor in an emergency situation: the ability to perform primary assessment of a seriously ill or injured patient, to determine the priority of the necessary actions, and to start treatment of all immediately life-threatening conditions [8]. The instrument contains three parts (formats). A case specific *checklist* with 8–11 critical decisions (‘recognizes airway obstruction, ‘supplies oxygen (NRM 12-15L/min.)’, scored yes/no for ‘correct’ and ‘timely’. Secondly, a *Competency Scale* (9 items, 6 on the ABCDE method (‘uses ABCDE approach on initial assessment’) and diagnostics (‘requests additional diagnostic studies’), 3 on communication (‘communicates with patient effectively’), rated on a 7-point scale, 1 = very weak, 7 = excellent). Thirdly, a *Global Performance Scale* using a single 10-point scale to rate ‘independent functioning in caring for acutely ill patients in the Emergency Department’ (10 = perfect). S1 Appendix provides an example. The pass/fail cut point is based on the Global Performance Scale (fail is <6). Cases were developed by experts and covered varying emergency care situations (case 1 Diabetic Ketoacidosis, case 2 Cerebrovascular accident, case 3 Hemorrhagic shock due to trauma, case 4 Burn victim).

The knowledge test contained 100 Multiple Choice (MC) items; the pass mark was 60%.

Inter-rater reliability was assessed using videotaped encounters, as has been done satisfactorily in previous investigations [5]. Using stratified random sampling, we selected 22 videos from the available 40 videos on two cases (1 and 4) for video assessments. The participants were stratified in 3 groups for both cases, based on their global performance: <7, between 7–8,>8. The amount of videos selected from the stratified groups was proportional to that in the group of participants (n = 144).

Eight raters from the regular assessor group viewed the 22 video registrations and assessed the candidates independently at a self-chosen time. All live and video based raters had different medical backgrounds (emergency medicine physician, surgeon, internist-intensivist or anaesthesiologist), as is typical in emergency medicine in clinical settings and for most emergency courses. They were all qualified instructors in internationally certified emergency medicine courses and trained according to international standards. The design and assessment of this type of emergency care training is comparable with ATLS (Advanced Trauma Life Support) and ALS (Advanced Life Support) courses.

### Measurements

Reliability of a test indicates the extent to which test results are reproducible and consistent under different conditions [9]–[11]. For validation, evidence was collected on different dimensions of construct validity: content, response process, internal structure (including reliability across items, stations, raters), relations with other variables and consequences [12]. We have measured: *content validity* (expert panel review of the assessment instrument), *response process* (debriefing sessions with raters), *internal structure* (factor analysis on the competency scale, analysis of internal consistency, calculation of agreement between ‘live’ and ‘videobased’ raters and a generalizability study to explore sources of error) and *relations with other variables* (associations between assessment formats).

### Statistical analysis

We used Cronbach's alpha to calculate internal consistency of scales. Inter-rater agreement was computed using the Intra Class Correlation (ICC) [11], [13]. We used the two-way random effects model of the ICC, as raters and candidates are random factors; ‘absolute agreement’ because this is a criterion-referenced test and ‘single measures’ because the assessment is normally done by a single rater. For the generalizability study, we used an absolute G-coefficient as both raters and cases can differ between candidates [14]. In order to analyze the reliability of the checklist, with a different number of critical decisions in both cases (8 and 7 items respectively for case 1 and 4), we used the mean summed score of both cases. Each candidate is assessed with one case, therefore case error could not be analyzed. The association between formats was calculated using Pearson's correlation coefficient.

The videotaped and non-videotaped groups were compared on main characteristics and results using the Student's t-test (independent and paired); one-way ANOVA was used to compare means of cases. We used SPSS version 22. The generalizability study was done using G String IV software.

### Ethics

All participants signed a consent form; the study was approved by the Dutch ethical board for research in (medical) education (NVMO, the Netherlands Association for Medical Education, approval no 210). The NVMO is an independent association that carries out activities for anyone involved in medical and health care education in the Netherlands and Flanders (Belgium).

## Results

### Characteristics of study subjects

144 residents, 80% of 179 course trainees, agreed to participate in this study. Of this group, 86 agreed to have their assessment videotaped (60%). Participants' average age was 29.3 years, 82% women, with an average of 5.6 months of experience with acute patients and an average score of 48% on the national knowledge test for family practitioners. The 22 residents participating in the videotaped assessments were on average 30 years, 68% women, had 4.6 months of experience with acute patients and a score of 47% on the national test. Differences between the sample and population were not statistically significant.

### Assessment results total group

The critical decisions on the checklist were scored as correct and timely for 80% of the residents (Table 1); means between cases were significantly different for the checklist (F (3, 80), p<.0001). Scores on the Competency Scale and the Global Performance Scale did not differ significantly between cases (p = 0.25/0.19). Thus, only the checklist showed variation between cases. Eight percent (n = 12) of the residents failed the assessment. On average 66% of the knowledge test items were answered correctly (SD = 9.78).

**Table 1:** Overview assessment results of the 3 parts of the skill assessment instrument (all cases).

			Cases		
Assessment format	Mean (SD)	1	2	3	4
n	144	44	26	36	38
Checklist critical decisions; Mean percentage correct and timely (7–11 items)	**80%**	92%	93%	40%	96%
(SD)	(29%)	(10%)	(9%)	(31%)	(13%)
Competency scale, 9 items, 7 pt (1 = poor, 7 = excellent) Mean	**5.61**	5.6	5.37	5.70	5.62
(SD)	(.70)	.56	.55	1.08	.50
Global Performance scale 1–10, 10 = positive end; Mean	**7.35**	7.44	6.87	7.44	7.47
(SD)	(1.24)	1.176	1.25	1.54	.93

### Assessment results of video based assessment

The 22 residents were individually (live) assessed by four different raters and all re-assessed by eight raters. Comparing live and video based assessment showed that the eight video based raters were more stringent than the live raters for all three parts of the assessment instrument (Table 2).

**Table 2:** Comparison video-based and live assessment results of 22 residents (case 1 and 4).

Assessment format	Live rating	Video based rating	P-value
Checklist; Mean percentage correct and timely (SD)	0.93 (0.12)	0.82 (0.11)	<0.001
Competency scale, Mean 9 items (SD)	5.50 (0.52)	5.30 (0.55)	0.012
Global Performance scale, Mean (SD)	7.3 (1.17)	6.9 (1.13)	0.002

### Content and response process evidence

The assessment instrument was evaluated by medical and educational experts and found to be representative of the emergency care skill to be assessed. The assessment instrument was implemented from Fall 2012 by the family practice training centre in the Netherlands. At the beginning of each assessment day, raters were briefed on the different scenarios and assessment instrument. During the assessment, a course director was available for questions. At the end of each assessment day a debriefing session was organized to evaluate the assessment process. Standardized patients were trained regularly.

### Internal structure

We found an indication of good coherence for the 9-item competency scale, as the factor analysis showed that 65% of the total variance in the items was explained by one factor. Inspection of the items loading on the first factor (1–6) indicated this factor could be summarized as ‘clinical competency’. Further analyses indicated a second factor, explaining another 9% of the variance. The second factor (item 7–9) could be summarized as ‘communication competency’. The two factors together explained 74% of the total variance (Factor analysis results are provided as S2 Appendix). The internal consistency (Cronbach's alpha), for the 9-item competency scale was 0.93, for the clinical skill subscale (item 1–6) 0.91, for the communication subscale (item 7–9) 0.86. The alpha for the knowledge test was 0.74. All scales had good internal consistency.

### Inter-rater reliability

The inter-rater agreement, indicated by the Intra Class Correlation (ICC), was poor for the dimensions ‘correct’ and ‘timely’ of the checklist (Table 3). The ICC on the clinical competency scale was moderate; it was poor for the communication competency scale. The ICC for the global Performance scale was moderate.

**Table 3:** IntraClass Correlation scores for the Checklist, Competency Scales and Global Performance Scale.

Assessment format	ICC score
Checklist (critical decisions)	
Correct (mean sum over 2 scenarios)	0.30
Timely (mean sum over 2 scenarios)	0.39
Clinical competency scale (item 1–6)	0.49
Communication competency scale (item 7–9)	0.27
Global Performance scale	0.50

Two way random effects model, absolute agreement, single measures, 95% confidence interval. ICC values 0.21–0.40 are considered ‘ poor/fair’, 0.40–0.60 ‘moderate’, 0.60–0.80 ‘substantial’, 0.80–1.00 ‘almost perfect’.

**Figure 1:**
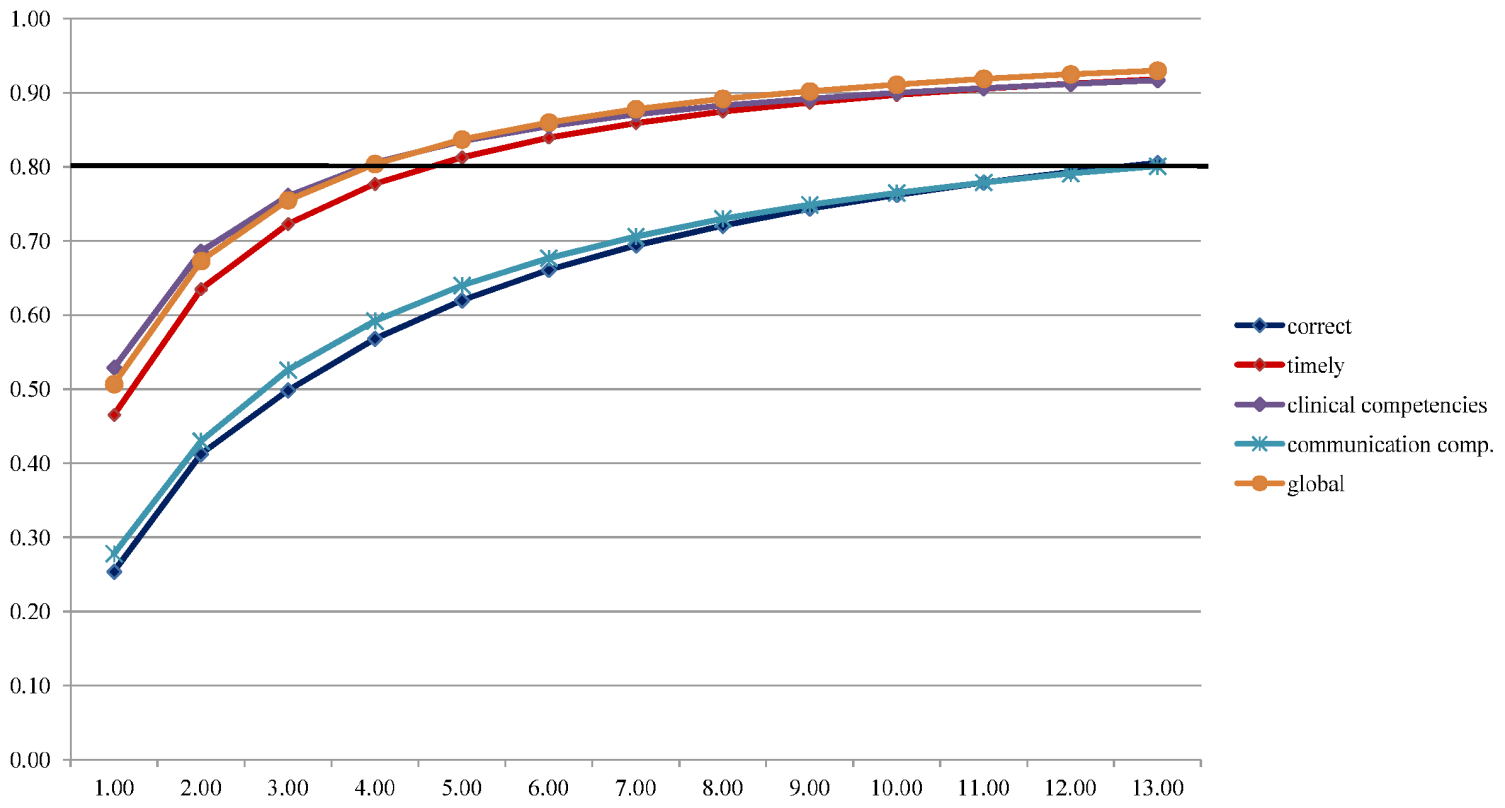
Absolute G-coefficients as a function of number of raters per assessment format.

Generalizability analysis showed that using the checklist, for the dimension ‘correct’ 13 raters would be required to achieve a reliability of 0.80 and for the dimension ‘timely’ 5 raters would be needed. For both the clinical competency scale and the global performance scale, 4 raters would be required to achieve an acceptable reliability (Fig. 1). Using the communication competency scale 13 raters would be needed.

To summarize, the 9-item competency scale showed good overall coherence and consisted of two factors (constructs): a clinical and communication subscale. Internal consistency of (sub)scales was high. The inter-rater agreement was moderate for the clinical competency and the global performance scale and poor for the communication scale and for the checklist (raters agree more on timeliness than on correctness of critical decisions). For a reliable assessment, 4 raters are needed using the clinical competency or global performance scale, 5-13 for the two dimensions of the checklist and 13 using the communication competency scale.

### Relation between assessment formats

The global performance scale was strongly correlated with the 9-item competency scale (0.85) and was less strongly, but still significantly correlated with the checklist (Table 4). The score on the knowledge test was correlated with the global performance scale and 9-item competency scale, but not with the checklist score.

**Table 4:** Pearson's Correlation coefficient between the 3 assessment formats and knowledge test.

		Competency Scale	Global Performance Scale	Knowledge test score
**Checklist score**	Correlation	.13	.19*	.14
	p	.12	.02	.10
	N	144	144	142
**Competency Scale**	Correlation		.85**	.24**
	p		.00	.004
	N		144	142
**Global Performance Scale**	Correlation			.27**
	p			.001
	N			142

(Correlation is significant at the 0.05 level* or the 0.01 level **, 2-tailed).

## Discussion

To the best of our knowledge, this is the first time a psychometric study has been done on three commonly used formats in emergency skills assessment, as part of a certified emergency care course.

It is clear that the weakest link in the assessment is the checklist, which has poor reliability. This may seem counter-intuitive, as case-specific, task oriented item lists were thought to minimize inter-rater variability, but various studies have shown this is not the case [15]. Our results are consistent with research in other domains spanning many years which has shown that checklists are not as objective as originally supposed [16], [17], have inferior validity, and do not show better reliability than global rating scales [18]–[21]. The dimension ‘timely’ in the checklist showed a higher reliability than the dimension ‘correct’. As timeliness is at the core of the emergency skill being assessed (the ABCDE approach is about the right sequence of actions), a higher level of agreement among raters on this dimension might be expected compared to agreement on the correctness of actions (e.g. whether the right tools were used). It is good to note that checklists are usually related to the correctness of clinical actions. Discussion with raters from our study on the poor inter-rater agreement with checklists made clear that they differed in their professional opinions on the relevance of the checklist items, probably a consequence of their varying medical background. The detailed character of checklists makes them more vulnerable to these differences than rating scales. Although we cannot completely rule out the possibility that our checklist was insufficiently validated, our findings are consistent with previous research and provide a legitimate base for our conclusion that checklists, in general, have insufficient reliability and validity and should be replaced by more global rating scales.

The use of checklists has grown particularly since the widespread implementation of Objective Structured Clinical Examinations (OSCEs), where clinical performance is scored at different stations with standardized problems [22], [23]. Checklists however tend to capture only whether some action occurred or not, whereas rating scales require the interpretation of actions [24]. In addition, checklists often do not capture increasing levels of expertise whereas rating scales do [25], [26]. An explanation might be that checklists reward thoroughness and mimic a novice's approach to problem solving, whereas expert clinicians are not necessarily thorough but highly accurate in problem solving [16], [18]. The intuitive attractiveness of checklists was confirmed in a study where clinicians found checklists more appropriate than competency scales to score clinical performance, but consistency in pass/fail decisions was equally poor for both forms [7]. Checklists with long lists of critical items to be scored often are labour-intensive and we found they are sensitive to case-differences, but do not add any reliability or validity to the assessment.

The competency scale and global performance scale showed high correlations; they seem to be measuring largely the same underlying dimensions. Advantages of the clinical competency scale are its potential for providing specific feedback to participants, which is useful for remediation. The communication scale showed poor reliability. Apparently raters agree more on clinical than on communication skills, possibly because they are less trained in assessing communication.

What would be the added value of the global performance scale? An advantage of this scale is the more holistic assessment of the candidate's performance, where competency scales are more focused on specific skills. Multi-case research will have to show which scales have higher inter-case reliability.

Video based raters appeared to be more stringent than live raters on all three parts of the assessment instrument. Raters assessing videos are more limited: they do not have the opportunity to ask supplementary questions or to alter their position; they might miss subtle checks by the candidate. On the other hand, they could feel more psychological distance to the candidate and hence look more objectively. We cannot conclude from this study which assessment is more valid, but in general delayed videos assessment of emergency skills is found as reliable as live assessment [27].

### Limitations

This study has some limitations. The assessment method studied was part of a specific emergency care course in The Netherlands. However, the course design and assessment method used are quite comparable to a number of internationally certified resuscitation courses, such as ATLS and ALS, and thus are recognizable for instructors from other courses. Another limitation of this study is the use of a single patient scenario per candidate. Extensive evidence in assessment research in the last decades shows content specificity is the main cause of unreliability and as such outweighs all other sources of bias(24,25). Various studies have shown that inter-rater reliability is a relatively small source of error compared with inter-case variability (which rarely exceeds a correlation of 0.1–0.3 across cases [24], [29], [30]). Thus, the reliability coefficients reported in this study may well be an upper limit of actual reliability when candidates see different cases. However, we have analyzed an internationally representative emergency care assessment situation where single patient scenario assessment is commonly used.

This study yields a number of practical implications for those involved in emergency care assessment.

A first implication is that assessment may be improved by putting more resources into multiple raters and multiple cases using the competency scales. While it is clear that additional raters will increase reliability, the data suggest that many raters will be needed to achieve acceptable standards. In fact, raters would be better deployed doing independent assessments with different scenarios, as performance of a candidate in one case is a poor predictor on another case [9]. One study showed that with 6 short (5 min.) cases and 1 rater per case a substantial reliability in emergency assessment can be achieved [28]. A challenge in strengthening reliability is to maintain validity, since complex skills require an integrated judgement and sufficient time to assess them [16]. Another way to increase the number of cases is to combine formative and summative assessment. During training, opportunities for observation and feedback can be used to collect more information on the candidate's performance with different scenarios. The formative value of assessment has been given increasing attention lately, characterized as a shift from assessment *of* learning to assessment *for* learning [24].

A second way to improve assessment would be to supplement performance testing with ‘know how’ test formats, using the strong relationship between ‘know how’ (applied knowledge) and ‘show how’ (demonstrate skills) for a variety of clinical procedural skills [31]. The ‘key feature’ test, a written test of clinical decision-making, may be useful. It requires short uncued answers, focusing on the critical steps (‘key features’) in a range of scenarios [16], [32]. ‘Key feature’ tests have modest correlation with clinical knowledge and competency tests [33]. Several studies have shown that these types of tests have good validity in predicting clinical performance in practice [34]–[36].

Although raters are typically experienced and trained, the low inter-rater agreement, also confirmed in other studies in general clinical skill assessment(6), shows there is room for improvement. Training has shown to have modest or no gains with experienced raters [37]; alignment sessions in which individual rating of videotaped performance is discussed may be more useful. Pass/fail decisions could be based on composite scores, using data from multiple tests and occasions [24].

## Conclusions

This study analyzed the validity of commonly used formats in the assessment of emergency skills, using checklists, competency scales and global performance scales in a single scenario assessment. One conclusion from this study is that checklists have poor validity and reliability in assessing emergency skills. As they are still standard practice in most assessments of emergency care courses, this is an important conclusion. Instead, the study suggests better measurement will result from using a clinical competency or a global rating scale. Nevertheless, we observed a limited overall agreement between raters in the assessment of emergency skills.

A concern is that the limited number of scenarios and raters usually applied in emergency care assessment, results in low reliability and validity. While additional raters may help, research should be conducted to examine the use of multiple cases in this assessment. Improving validity of assessment is vital, considering the worldwide use of emergency care training, the implications for patients, and the high costs of training. More research into improving valid and feasible assessment of emergency skills is strongly recommended, using several (short) scenarios and raters.

## Supporting Information

S1 Appedix **Assessment form emergency care skill.**
(DOCX)

S2 Appendix **Factor analysis results.**
(DOCX)
