# Supplementary material for: Assessing the Assessment in Emergency Care Training
Source: PLoS One. 2014 Dec 18;9(12):e114663. doi: 10.1371/journal.pone.0114663 (PMC4270684; doi:10.1371/journal.pone.0114663)
Supplement: S1 Appedix — Assessment form emergency care skill. (DOCX) [file pone.0114663.s001.docx]

**Appendix S1: Assessment instrument emergency care skill** (for scenario 1:Diabetic Ketoacidosis)

Name candidate: ………………………………. Number: ……..

Date: …………………………………………… Rater:……………………………………….

| **Checklist (items on critical decisions)** | | | | **Executed** | | | | | | |  |  |  |
| --- | --- | --- | --- | --- | --- | --- | --- | --- | --- | --- | --- | --- | --- |
|  |  |  |  | **correct** | | | | **timely** | | |  |  |  |
| **A** | | Recognizes airway obstruction (snoring breathing) | |  | | | |  | | |  |  |  |
|  |  | Treats airway obstruction (manual maneuver) | |  | | | |  | | |  |  |  |
| **B** | | Measures breathing frequency | |  | | | |  | | |  |  |  |
|  |  | Supplies oxygen (NRM 12-15 L/min.) | |  | | | |  | | |  |  |  |
| **C** | | Recognizes shock (pulse 104/min and BP 90/60 mmHg) | |  | | | |  | | |  |  |  |
|  |  | Starts intravenous fluid bolus (Normal saline 0.9% of Lactated Ringer’s) | |  | | | |  | | |  |  |  |
| **D** | | Measures glucose | |  | | | |  | | |  |  |  |
|  |  | Assesses state of consciousness using GCS parameters | |  | | | |  | | |  |  |  |
| **E** | |  | |  | | | |  | | |  |  |  |
|  | | **Competency Scale** | | **very weak** | | **weak** | **insufficient** | **questionable** | | **sufficient** | **good** | | **excellent** |
|  | | **ABCDE approach** | |  | |  |  |  | |  |  | |  |
| 1 | | Uses ABCDE approach on initial assessment | |  | |  |  |  | |  |  | |  |
| 2 | | Uses ABCDE approach on initial treatment | |  | |  |  |  | |  |  | |  |
| 3 | | Uses re-assessment properly | |  | |  |  |  | |  |  | |  |
|  | | **Additional actions** | |  | |  |  |  | |  |  | |  |
| 4 | | Requests additional diagnostic studies | |  | |  |  |  | |  |  | |  |
| 5 | | Proposes a working diagnoses | |  | |  |  |  | |  |  | |  |
| 6 | | Consults specialist when needed | |  | |  |  |  | |  |  | |  |
|  | | **Communication** | |  | |  |  |  | |  |  | |  |
| 7 | | Communicates with patient effectively | |  | |  |  |  | |  |  | |  |
| 8 | | Gives clear instructions to nurse | |  | |  |  |  | |  |  | |  |
| 9 | | Radiates a calm and confident attitude | |  | |  |  |  | |  |  | |  |

**Global Performance Scale
Assessment of independent function in caring for acutely ill patients in the Emergency Department**:… (1-10)

**Passed/Failed**
