# Supplementary material for: Assessing the Assessment in Emergency Care Training
Source: PLoS One. 2014 Dec 18;9(12):e114663. doi: 10.1371/journal.pone.0114663 (PMC4270684; doi:10.1371/journal.pone.0114663)
Supplement: S2 Appendix — Factor analysis results. (DOCX) [file pone.0114663.s002.docx]

**Appendix S2. Principal Component Analyses of Competency scale**

| **Communalities** | | |
| --- | --- | --- |
| Competency Scale | Initial | Extraction |
| 1 uses ABCDE approach on initial assessment | 1.000 | .637 |
| 2 uses ABCDE approach on initial treatment | 1.000 | .768 |
| 3 uses re-assessment properly | 1.000 | .790 |
| 4 requests additional diagnostics | 1.000 | .711 |
| 5 proposes a working diagnoses | 1.000 | .610 |
| 6 consults specialist when needed | 1.000 | .702 |
| 7 communicates with patient effectively | 1.000 | .869 |
| 8 gives clear requests to nurse | 1.000 | .814 |
| 9 radiates a calm and secure attitude | 1.000 | .749 |
| Extraction Method: Principal Component Analysis. | | |

| **Total Variance Explained** | | | | | | | | | |
| --- | --- | --- | --- | --- | --- | --- | --- | --- | --- |
| Component | Initial Eigenvalues | | | Extraction Sums of Squared Loadings | | | Rotation Sums of Squared Loadings | | |
|  | Total | % of Variance | Cumulative % | Total | % of Variance | Cumulative % | Total | % of Variance | Cumulative % |
| 1 | 5.845 | 64.948 | 64.948 | 5.845 | 64.948 | 64.948 | 3.875 | 43.061 | 43.061 |
| 2 | .805 | 8.943 | 73.891 | .805 | 8.943 | 73.891 | 2.775 | 30.830 | 73.891 |
| 3 | .591 | 6.572 | 80.463 |  |  |  |  |  |  |
| 4 | .437 | 4.850 | 85.313 |  |  |  |  |  |  |
| 5 | .371 | 4.119 | 89.432 |  |  |  |  |  |  |
| 6 | .290 | 3.220 | 92.653 |  |  |  |  |  |  |
| 7 | .245 | 2.723 | 95.376 |  |  |  |  |  |  |
| 8 | .226 | 2.516 | 97.891 |  |  |  |  |  |  |
| 9 | .190 | 2.109 | 100.000 |  |  |  |  |  |  |
| Extraction Method: Principal Component Analysis. | | | | | | | | | |

| **Component Matrix^a^** | | |
| --- | --- | --- |
| Competency Scale | Component | |
|  | 1 | 2 |
| 1 uses ABCDE approach on initial assessment | .763 | -.237 |
| 2 uses ABCDE approach on initial treatment | .824 | -.297 |
| 3 uses re-assessment properly | .878 | -.137 |
| 4 requests additional diagnostics | .830 | -.151 |
| 5 proposes a working diagnoses | .778 | -.062 |
| 6 consults specialist when needed | .782 | -.300 |
| 7 communicates with patient effectively | .750 | .554 |
| 8 gives clear requests to nurse | .824 | .368 |
| 9 radiates a calm and secure attitude | .816 | .289 |
| Extraction Method: Principal Component Analysis. | | |
| a. 2 components extracted. | | |

| **Rotated Component Matrix^a^** | | | | | |
| --- | --- | --- | --- | --- | --- |
|  | | | | Component | |
|  |  |  |  | 1 | 2 |
| 1 uses ABCDE approach on initial assessment | | | | .743 | .292 |
| 2 uses ABCDE approach on initial treatment | | | | .829 | .284 |
| 3 uses re-assessment properly | | | | .771 | .442 |
| 4 requests additional diagnostics | | | | .742 | .401 |
| 5 proposes a working diagnoses | | | | .647 | .438 |
| 6 consults specialist when needed | | | | .798 | .255 |
| 7 communicates with patient effectively | | | | .239 | .901 |
| 8 gives clear requests to nurse | | | | .413 | .802 |
| 9 radiates a calm and secure attitude | | | | .456 | .735 |
| Extraction Method: Principal Component Analysis. Rotation Method: Varimax with Kaiser Normalization. a. Rotation converged in 3 iterations. | | | | | |
|  | | | | | |
| **Component Transformation Matrix** | | |  |  |  |
| Component | 1 | 2 |  |  |  |
| 1 | .781 | .625 |  |  |  |
| 2 | -.625 | .781 |  |  |  |
